# Author's Reply. RE: An intercultural perspective towards supporting antipsychotic medication adherence in clinical practice

**DOI:** 10.1192/bjb.2023.52

**Published:** 2023-08

**Authors:** Tharun Zacharia

**Affiliations:** Consultant Psychiatrist, South London and Maudsley NHS Foundation Trust, UK. Email: tharun.zacharia@slam.nhs.uk

Thank you for your thought-provoking response on 24 April 2023. I found your agreed emphasis on humility, genuineness and person-centeredness in the cultural learning within the clinician–patient relationship to be truly refreshing. By positioning clinicians as active learners, equalising the power dynamic and centralising the therapeutic alliance, the proposed approach aligns well with the principles of patient-centred care.

The discussion of how such an approach could be practically integrated into routine history-taking and other aspects of clinical care is important but, in my opinion, should not be stated too prescriptively as to stifle the naturally forming alliance between doctor and patient. The individual background and traits of the clinician are likely to affect the approach and, therefore, a ‘clinician-centred’ approach is required. Broadly speaking though, the introduction of cultural competence training in medical schools to promote engagement with these cultural aspects of the clinical–patient dynamic is vital, allowing the doctor to reflect on how their own culture and that of the patient can be used to strengthen the alliance.^1^

I agree that Balint groups are a suitable opportunity to address issues of race, ethnicity and culture more freely. At GKT Medical School (King's College London), Balint groups are incorporated into medical student training (third year) and, through my experience as a co-facilitator of these groups, I have found medical students to often feel liberated by the unique opportunity to speak about the above issues in a reflective context while beginning clinical placements.

I share your concern about potentially overemphasising medication adherence as a standalone goal, especially when considering individuals from minority ethnic backgrounds. However, evidence clearly suggests the efficacy of antipsychotic medication.^2^ As this is an evidence-based treatment, it's important that we consider parity of care among ethnic minorities, and therefore the article promotes important discussion about tailoring a person-centred approach in this particular intervention. I agree, however, that more work is also needed to address non-pharmacological interventions.

The study you referenced by Mclean et al^3^ sheds light on the disparities in mental healthcare, indicating a tendency toward overmedication and a lack of psychotherapy options for these patients. It is crucial to take into account alternative explanatory models of illness, including psychological and socioeconomic factors, in order to provide a comprehensive understanding of mental health and optimise patient outcomes. By incorporating these perspectives, we can avoid perpetuating a narrow biomedical model that has sometimes dominated Western psychiatry to the detriment of patients.^3^

I agree with your appreciation of the therapeutic alliance as a fundamental component of effective care. Your point about a good therapeutic alliance serving as a vehicle towards improvement, independent of medication, aligns with growing recognition of the multifaceted nature of mental health treatment. For many patients, medication will remain an important aspect of achieving an optimal outcome.^4,5^

Sadly, I agree a stigma often persists around foreign-born or foreign-trained doctors and medical leaders. A culture change within many National Health Service organisations is required. Cultural competence training, brave leadership and trust-wide culture-based reflective forums may be a starting point to open these discussions in a non-judgemental setting. We already recognise that foreign-trained doctors perform significantly worse in the CASC membership examination after controlling for educational and background variables.^6^ This perpetuates the idea that cultural differences in the clinician are unvalued.

Thank you once again for your thought-provoking letter, which highlights the significance of intercultural perspectives in mental healthcare. Your insights and recommendations serve as valuable contributions to the ongoing dialogue surrounding the provision of patient-centred and culturally sensitive care.
